# Gingival bleeding and calculus among 12-year-old Chinese adolescents: a multilevel analysis

**DOI:** 10.1186/s12903-020-01125-3

**Published:** 2020-05-19

**Authors:** Hong Chen, Rui Zhang, Ran Cheng, Ting Xu, Tao Zhang, Xiao Hong, Xing Zhao, Yunyun Wu, Li Cheng, Tao Hu

**Affiliations:** 1 Graduate Student in Department of Preventive Dentistry, West China School & Hospital of Stomatology, Sichuan University, Chengdu, China; 2 State Key Laboratory of Oral Diseases, Sichuan University, Chengdu, PR China; 3 Associate Professor in Department of Preventive Dentistry, West China School of Stomatology, Sichuan University, Chengdu, China; 4 Professor in Department of Preventive Dentistry, West China School of Stomatology, Sichuan University, Chengdu, Sichuan PR China; 5 West China School of Public Health, Sichuan University, Chengdu, China

**Keywords:** Oral health, Gingival bleeding, Dental calculus, Adolescents, Cross-sectional study

## Abstract

**Background:**

Gingivitis is a common oral health problem, and untreated gingivitis can progress to periodontitis. The objectives of this study were to (1) explore associated factors of gingival bleeding and calculus among 12-year-old adolescents; (2) find predictive models for gingivitis management.

**Methods:**

Four thousand five hundred twenty-five subjects aged 12 in Sichuan Province were investigated. The questionnaire and clinical examination were applied in schools, and two-level logistic regression models were constructed to interpret the effect of individual and contextual factors on Chinese adolescents’ gingival bleeding and calculus.

**Results:**

46.63% (95%CI: 40.71, 51.60) and 66.94% (95%CI: 56.85, 67.45) of the subjects presented gingival bleeding and calculus, respectively. For the gingival bleeding cases, the model showed the significant associated indicators were *hukou* (OR = 0.61, 95% CI: 0.52–0.72), family size (OR = 1.41, 95% CI: 1.19–1.68), parental educational level (father: OR = 0.53, 95% CI: 0.45–0.63; mother: OR = 0.71, 95% CI: 0.59–0.86), tooth-brushing frequency (OR = 0.35, 95% CI: 0.26–0.48), dental floss use (OR = 0.58, 95% CI: 0.41–0.83), sugar-containing drink consumption (OR = 2.11, 95% CI: 1.80–2.49), and dental visit (OR = 1.44, 95% CI: 1.19–1.74). It also confirmed that gender (OR = 1.32, 95% CI: 1.13–1.54), *hukou* (OR = 0.69, 95% CI: 0.59–0.82), family size (OR = 1.34, 95% CI: 1.12–1.59), parental educational level (father: OR = 0.46, 95% CI: 0.39–0.54; mother: OR = 0.65, 95% CI: 0.59–0.82), tooth-brushing frequency (OR = 0.57, 95% CI: 0.42–0.78), dental floss use (OR = 0.66, 95% CI: 0.48–0.90) and sugar-containing drink consumption (OR = 1.30, 95% CI: 1.11–1.53) were associated factors for dental calculus.

**Conclusions:**

Gingival bleeding and calculus were common in western Chinese adolescents. Socio-demographic factors including gender, *hukou* and family factors are strong determinants of gingival health in Chinese adolescents. In addition, health-related lifestyle behaviors such as healthy diet, good hygiene care and more dental visits are good predictors of better gingival status.

## Background

Oral diseases are the most common noncommunicable diseases, and have become alarming public health problems worldwide [1]. Gingivitis, one of the most common oral health problems, is a mild form of periodontal disease [1, 2]. Plaque-induced gingivitis is considered to be the most prevalent type of gingivitis [3, 4]. The clinical symptoms of gingivitis include redness, oedema and bleeding at the gingival margin [5]. During puberty (girls, 11–13 years; boys, 13–14 years), periodontal tissues may have an amplified response to local factors: dental plaque, calculus, food debris, and materia alba [6]. And regular removal of these local factors can prevent the occurrence and progression of early gingival disease [7, 8].

Dental calculus (mineralized biofilms), located along the gingival margin, is an important gingivitis-contributing factor [9, 10]. Calculus can’t be removed easily from the surface of teeth and provides a substratum for plaque retention in the vicinity to the gingiva [9, 10]. Calculus can also be a surrogate indicator of long-term exposure to biofilm and poor oral hygiene practices [11]. Its presence, along with gingival inflammation, is associated with the initiation and progression of early-onset periodontitis [10].

Gingivitis and periodontitis can be considered as a continuum of the same inflammatory process, and untreated gingivitis can progress to periodontitis complicated with further tissue destruction and bone reabsorption [2, 3, 12]. Therefore, gingivitis management of adolescents is a prevention strategy for advanced periodontal diseases that might persistently play a crucial role in reducing the periodontal disease burden [2, 4]. The greatest share of oral health problems is attributable to the social conditions in which people live and work, referred to as the social determinants of health (SDH) [13–15]. There are huge disparities in the level of economic development and the availability of healthcare resources in China [16]. The contextual differences are associated with several clinical outcomes of various oral diseases and could thus be related to gingivitis. Other individual SDH factors associated with gingivitis include sex and ethnic inequalities [13, 14], family factors [14, 17], and health behaviors [14, 17, 18]. Therefore, it is important to identify associated individual and contextual factors whose modification can reduce the prevalence of gingival bleeding and calculus in the future, and further, to provide advises on controlling such factors for maintaining a good quality of life [19].

Traditional periodontal examinations are resource-intensive, and a large-scale screening for adolescents is infeasible in China [20]. Recently, self-reported questionnaires have been proposed to predict the probability in periodontal surveys. And the validation of prediction models based on self-reported questionnaires were demonstrated in Taiwan [20], Australia [21], Germany [22], Japan [23], and the United States [24]. Twelve years has been recommended by World Health Organization (WHO) as the indicator age group for international benchmarking of adolescents’ oral health [25, 26]. Little is known about the prevalence of gingival bleeding and calculus in adolescents with large sample size in western China. Therefore, the present study aimed to investigate the associated factors for gingival bleeding and dental calculus in 12-year-old adolescents and to build predictive models for gingivitis management and treatment planning.

## Methods

### Study design

This cross-sectional study was a part of the Fourth National Oral Health Survey in China [27–29]. It was a school-based survey conducted from 07/01/2015 to 09/17/2016. Ethics approval was obtained from the Stomatological Ethics Committee of the Chinese Stomatological Association and the Ethics Committee of West China Hospital of Stomatology, Sichuan University (Approval No. 2014–003).

### Survey sampling

**Fig. 1:**
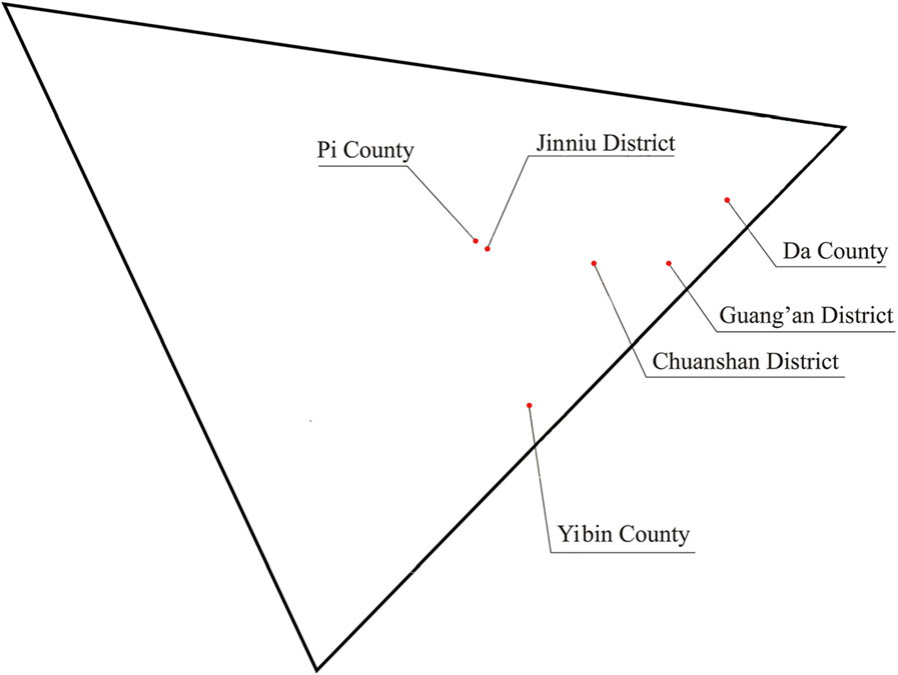
The schematic diagram of Sichuan Province

The target population was selected from about 1, 018, 500 adolescents at the age of 12 years in Sichuan Province. Sichuan Province is located in western China and represents one of the largest provinces with regard to area and population [27, 28]. A complex, multi-stage sampling design [27–29] was used to select participants who were representative of the province’s population. In the first stage, six regions (Guang’an District, Chuanshan District, Jinniu District, Da County, Yibin County and Pi County) were obtained from 181 regions with probability-proportional-to-size (PPS) method (Fig. 1). And then a random sampling of schools was selected in the second stage in each region selected in the first stage [27, 28]. In the third stage, a computer-generated random number method was used for examinees selection at the aged 12 years [27].

A letter of invitation and informed consent form were sent to the guardian of potential participants firstly, emphasizing that the participation was voluntary. Participants and their statutory guardians were required to sign informed consent forms. Adolescents with serious physical or psychological illness or disadvantages, who were unable or unwilling complete the examination and questionnaire, were excluded. Ultimately, a random sample of 4525 students completed the survey; this number was greater than the expected 4420 participants calculated by the formula:

$$ \mathrm{n}= deff\frac{\mu^2\left(1-p\right)}{\varepsilon^2p\left(1- nonresponse\right)} $$

in which the design efficiency *deff* = 2.5 [27, 28], the confidence of investigation *μ* = 1.96, the acceptable error *ε* = 15%, the prevalence of caries in people at aged 12 years from the Third National Oral Health Survey in China *p* = 28.9% [30] and the non-response rate was 5%. Of all, gender parity and rural/urban parity would be required [27, 28]. The gingival bleeding and calculus prevalence rates were 57.7 and 59.0%, respectively [30]. Therefore, respective smaller sample sizes (*N* ≤ 1318 and *N* ≤ 1249) would be calculated.

### Questionnaire

The questionnaire was distributed to 4800 participants in 12 years old in Sichuan Province. Every participant was asked to fill an interviewer-administered questionnaire with the guidance of trained dental students in their schools. The independent variables were composed of individual (intermediate SDH) and contextual (regions) factors associated with health (Table 1) [13–15]. These variables were adopted by the World Health Organization Commission on Social Determinants of Health (CSDH). The correlations between gingival status (gingival bleeding and calculus) and Individual factors (gender, ethnicity, *hukou*, family size, parental educational level, and health-related lifestyle behaviors) [14–17] and contextual factors (regions) were assessed [14]. Health-related lifestyle behaviors were assessed using oral hygiene behaviors (toothbrushing frequency and dental floss usage), diet habits (sugar consumption), dental visit, and dental education [14, 15]. The independent variables were categorized according to previous studies [14–17]. Contextual variable was the region-level difference. And the 6 regions represented different levels of urbanization [27, 28]. The urbanization that differs “between” regions was a value from 0 to 1. And 6 selected regions were distributed into 3 urbanization levels: high, middle and low levels, two for each level [27, 28].

**Table 1:** Independent variables according to analysis level and SDH categories

Level	Classification	Variable	Description and categories
1st Level—Individual	Intermediate SDH	Gender	Sex of individual, Female/Male
		Ethnicity	Han/Others
		*hukou*	Rural/Urban
		Family size	Number of Children, One child/More than one child
		Father’s educational level	Schooling of father, Middle school or lower/More than middle school
		Mother’s educational level	Schooling of parents, Middle school or lower/More than middle school
		Toothbrushing frequency	Never/Sometimes/1/≥2
		Dental floss usage	Never/Yes (≥0)
		Candy/chocolate /cookies /cakes	≤1/week/> 1/week
		Sugar-containing soft drink/soda/ milk/yogurt /tea/coffee/water	≤1/week/> 1/week
		Dentist visit during past year	> 0/0
		Frequency of dental education (previous semester)	> 0/0
2st Level—Contextual	Structural SDH	Regions represent different levels of urbanization	Guang’an District, Chuanshan District, Jinniu District, Da County, Yibin County and Pi County

*Abbreviations*: *SDH* social determinants of health

### Quality control

Quality control was conducted as following: three licensed dentists, who had worked for more than 2 years and cooperated with three recorders, were trained by a standard examiner (the fourth examiner) before beginning this survey. No probing of pocket depths was performed, so Cohen’s Kappa statistic was used to assess the inter-examiner variability of dental caries in adolescents and probing pocket depths in adults. The final Kappa scores obtained on inter- or intra-examiner variability were ≥ 0.8. Furthermore, the inter-examiner variability in three regions during the survey were ≥ 0.8 as well.

### Clinical assessment

All clinical procedures were conducted in the adolescents’ school in the following sequence. Each participant in the selected regions received an oral health examination by a trained licensed dentist according to the criteria issued by the WHO [25, 31]. The portable equipment used consisted of a dental chair, external light source, plane mouth mirrors, air compressor and the community periodontal index probe (WHO/CPI probe) in conjunction with WHO clinical criteria and visual examinations [25, 32]. Gingival bleeding was defined as the presence of gingival bleeding upon gentle probing (BOP) in at least one site [25]. The gingival calculus was explored by using CPI probe. The probe started just distal to the midpoint of the buccal surface and then gently moved into the mesial interproximal area. The same procedure was completed on the palatal surface. Bleeding sites were scored after the sites of a single quadrant were probed. Each site was scored as no bleeding = 0 and bleeding = 1. A gentle tactile exam was used to locate calculus deposits. Each site was scored as followings: no calculus = 0; calculus = 1. Missing (not yet erupted, or extracted) or excludable (dental caries, extensive restorations or orthodontic bands or brackets) were not evaluated [4]. Dental plaque, probing depth, and clinical attachment level were not assessed. No radiographic examination was performed.

After completing the oral examination, all adolescents received an oral evaluation form to take home. This form classified the child’s oral health status according to the severity of the oral findings and recommended the timing of their next dental visit [4, 25]. Those adolescents with calculus would be recommended that they “must visit a dentist as soon as possible”.

### Statistical analysis

The data were independently extracted for the statistical analyses by two of the authors (H. C. and R. Z.). The whole information was extracted from the questionnaires and the oral health assessment form. EpiData Version 3.1 (EpiData Association, http:// www.epidata.dk, Epidata Association, Odense, Den- mark) was used for data capture. In order to ensure the consistence and the accuracy of the data, the items were assessed more than 3 times during the input process. Any disagreements or mistakes were assessed further and dealt by the original data in questionnaires.

The simple validation method was used here, and the predictive models were built. Three-quarters of the data were randomly selected and used as training data to develop two-level (first level: individuals; second level: regions) logistic regression models with random intercepts. Firstly, univariate analysis (Pearson’s Chi-square test) was used to select factors that might be associated with the outcome variables. After that, predictive models were established with the selected variables mentioned above for gingival bleeding and calculus respectively. The *p*-value, odds ratio (OR) and 95% confidence intervals (95% CI) were estimated to specify the predictive models, and the variables with *p*-value didn’t reach 0.05 were removed from the models [33]. The variance partition coefficient (VPC) was calculated to measure the percent of the total variance (summed across the individual level and all contextual levels included in the model) attributable to a given contextual level [34]. Finally, the other one-quarter data (the remaining data) were used for model validation.

All descriptive analyses were performed with the software IBM SPSS Statistics v. 19.0 (IBM Corp., Armonk, NY, USA). The other statistical analyses were implemented by using the lme4 package and Threshold ROC package in *R* version 2.13.1 (*R* Foundation for Statistical Computing, Vienna, Austria).

## Results

A total of 4525 subjects aged 12 years were selected for eligibility in this study with an effective response rate of 94.27%. Among the participants, 48.60% (*n* = 2199) of the participants were boys, and 51.40% (*n* = 2326) were girls. The prevalence of gingival bleeding was 46.63% (*n* = 2110; 95%CI: 40.71, 51.60), that of calculus was 66.94% (*n* = 3029; 95%CI: 56.85, 67.45). The mean scores of the individual scores of gingival bleeding and calculus were 4.00 and 4.57, respectively.

**Fig. 2:**
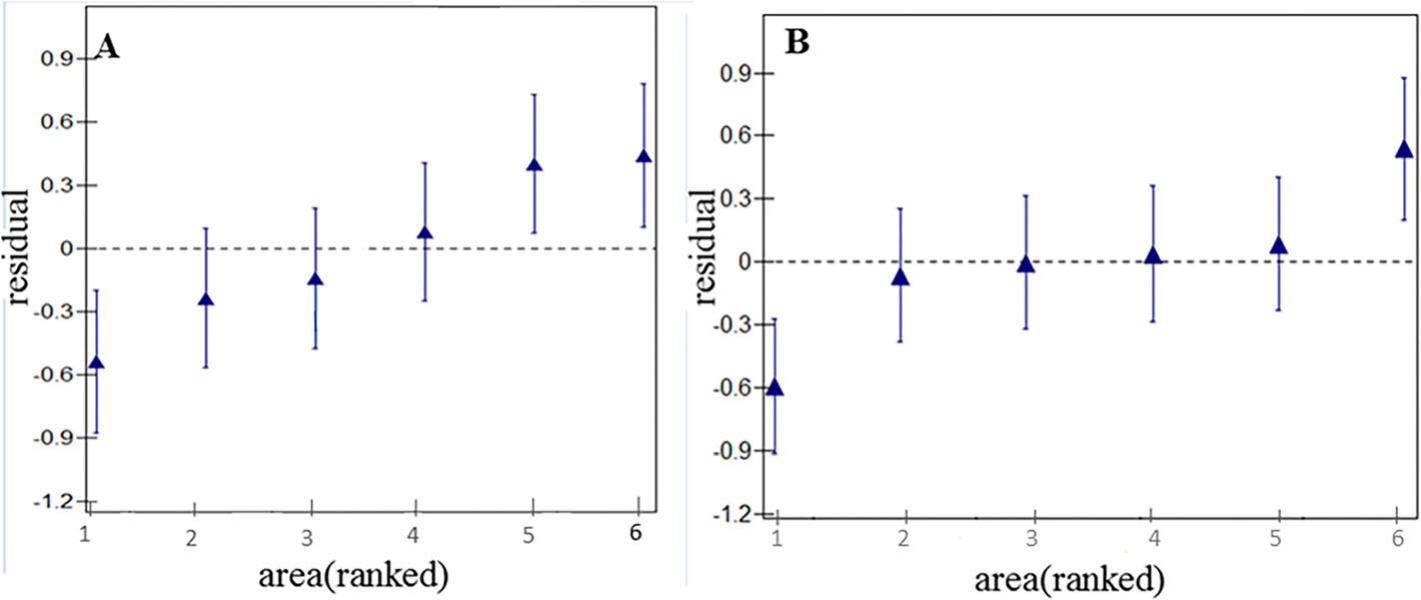
Plots of regions residuals with 95%CIs. (**a**) gingival bleeding and (**b**) calculus. **a**: sequencing from left to right: Jinniu District, Guang’an District, Yibin County, Da County, Chuanshan District and Pi County. **b**: sequencing from left to right: Yibin County, Da County, Guang’an District, Jinniu District, Chuanshan District and Pi County

The details of the total data were showed in Table 2. Among, the training dataset characteristics and the results of univariate analyses were presented in Table 3. As Fig. 2 showed, gingival bleeding in three regions (Guang’an District, Jinniu District, and Pi County) and calculus in two (Yibin County, and Pi County) indicated significant variations (ie, they did not overlap the horizontal line at zero). Therefore, two-level logistic regression models were built with the selected variables by univariate analyses.

**Table 2:** Descriptive characteristics of the participants (*N* = 4525)

	Number	Percent	95% CI	
**Gingival bleeding**	2110	46.63	45.18	48.08
**Calculus**	3029	66.94	65.57	68.31
**Areas**				
Pi County	590	13.04	12.06	14.02
Chuanshan District	810	17.90	16.78	19.02
Da County	838	18.52	17.39	19.65
Yibin County	845	18.67	17.54	19.81
Jinniu District	682	15.07	14.03	1.11
Guang’an District	760	16.80	15.71	17.89
**Gender**				
Female	2326	51.40	49.95	52.86
Male	2199	48.60	47.14	50.05
***hukou***				
Rural	2320	51.27	49.81	52.73
Urban	2205	48.73	47.27	50.19
**Ethnicity**				
Han	4475	98.90	98.59	99.20
Others	50	1.10	2.80	1.41
**Family size**				
One child	2883	63.71	62.31	65.11
More than one child	1642	36.29	34.89	37.69
**Father’s educational level**				
Middle school or lower ^a^	2960	65.41	64.03	66.80
More than middle school^b^	1565	34.59	33.20	35.97
**Mother’s educational level**				
Middle school or lower ^a^	3314	73.24	71.95	74.53
More than middle school^b^	1211	26.76	25.47	28.05
**Toothbrushing frequency**				
Never	329	7.27	6.51	8.03
Sometimes	670	14.81	13.77	15.84
1	1925	42.54	41.10	43.98
≥ 2	1601	35.38	33.99	36.77
**Dental floss usage**				
Never	4273	94.43	93.76	95.10
Yes	252	5.57	4.90	6.24
**Candy/chocolate /cookies /cakes**				
≤ 1/week	1820	40.22	38.79	41.65
> 1/week	2705	59.78	58.35	61.21
**Sugar-containing soft drink/soda/ milk/yogurt /tea/coffee/water**				
≤ 1/week	2999	66.28	64.90	67.65
> 1/week	1526	33.72	32.35	35.10
**Dentist visit during past year**				
> 0	991	21.90	20.70	23.11
0	3534	78.10	76.89	79.30
**Frequency of dental education (previous semester)**				
> 0	537	11.87	10.92	12.81
0	3988	88.13	87.19	89.08

*Abbreviation*: ^a^Middle school or lower, ≤9 years; ^b^More than middle school, >9 years

**Table 3:** Variables associated with gingival bleeding and calculus using training data as selected by univariate analysis (*n* = 3394)

Variables	Categories	Gingival bleeding				Calculus			
		Yes	No	OR (95%CI)	***p***	Yes	No	OR (95%CI)	***p***
**Region**	Pi County	264	190	1		355	99	1	
	Chuanshan District	318	276	0.83 (0.65, 1.06)	0.14	402	192	**0.58 (0.44, 0.77)**	**< 0.01**
	Da County	319	323	**0.71 (0.56, 0.91)**	**0.01**	439	203	**0.60 (0.46, 0.79)**	**< 0.01**
	Yibin County	315	300	**0.76 (0.59, 0.96)**	**0.02**	381	234	**0.45 (0.34, 0.60)**	**< 0.01**
	Jinniu District	137	376	**0.26 (0.20, 0.34)**	**< 0.01**	310	203	**0.43 (0.32, 0.56)**	**< 0.01**
	Guang’an District	213	363	**0.42 (0.33, 0.54)**	**< 0.01**	373	203	**0.51 (0.39, 0.68)**	**< 0.01**
**Gender**	Female	805	949	1		1112	642	1	
	Male	761	879	1.02 (0.89, 1.17)	0.77	1148	492	**1.35 (1.17, 1.56)**	**< 0.01**
**Ethnicity**	Others	20	19	1		31	8	1	
	Han	1546	1809	0.81 (0.43, 1.53)	0.52	2229	1126	0.51 (0.22, 1.06)	0.09
***hukou***	Rural	938	809	1		1248	499	1	
	Urban	628	1019	**0.53 (0.46, 0.61)**	**< 0.01**	1012	635	**0.64 (0.55, 0.74)**	**< 0.01**
**Family size**	One child	982	1178	1		1420	740	1	
	More than one child	584	650	1.08 (0.94, 1.24)	0.30	840	394	1.11 (0.96, 1.29)	0.17
**Father’s educational level**	Middle school or lower ^a^	1168	1045	1		1624	589	1	
	More than middle school^b^	398	783	**0.45 (0.39, 0.53)**	**< 0.01**	636	545	**0.42 (0.36, 0.49)**	**< 0.01**
**Mother’s educational level**	Middle school or lower ^a^	1250	1240	1		1756	734	1	
	More than middle school^b^	316	588	**0.53 (0.46, 0.62)**	**< 0.01**	504	400	**0.53 (0.45, 0.62)**	**< 0.01**
**Brushing frequency (times/day)**	Never	148	111	1		188	71	1	
	Sometimes	348	142	**1.84 (1.34, 2.52)**	**< 0.01**	399	91	**1.66 (1.16, 2.36)**	**0.01**
	1	739	711	0.78 (0.60, 1.02)	0.07	1013	437	0.88 (0.65, 1.17)	0.38
	≥2	331	864	**0.29 (0.22, 0.38)**	**< 0.01**	660	535	**0.47 (0.34, 0.62)**	**< 0.01**
**Dental floss use**	Never	1512	1698	1		2161	1049	1	
	Yes	54	129	**0.47 (0.33, 0.64)**	**< 0.01**	99	85	**0.56 (0.42, 0.76)**	**< 0.01**
**Candy/chocolate /cookies /cakes**	≤1/week	594	786	1		907	473	1	
	> 1/week	972	1042	**1.23 (1.08, 1.42)**	**< 0.01**	1353	661	1.07 (0.92, 1.23)	0.38
**Sugar-containing soft drink/soda/ milk/yogurt /tea/coffee/water**	≤1/week	377	764	1		701	440	1	
	> 1/week	1189	1064	**2.26 (1.95 2.63)**	**< 0.01**	1559	694	**1.41 (1.21, 1.64)**	**< 0.01**
**History of dental visit (previous year)**	No	1224	1436	1		1779	881	1	
	Yes	342	392	1.02 (0.87, 1.21)	0.78	481	253	0.94 (0.79, 1.12)	0.49
**Frequency of dental curricula (previous semester)**	0	1424	1569	1		2020	973	1	
	≥1	142	259	**0.60 (0.49, 0.75)**	**< 0.01**	240	161	**0.72 (0.58, 0.89)**	**< 0.01**

*Abbreviations*: ^a^Middle school or lower: ≤9 years; ^b^More than middle school: >9 years

Table 4 presented the eight individual variables significantly associated with gingival bleeding (*p* < 0.05) in two-level logistic regression analysis. The model for gingival bleeding was established including *hukou* (OR = 0.61, 95% CI: 0.52–0.72), family size (OR = 1.41, 95% CI: 1.19–1.68), parental educational level (Father: OR = 0.53, 95% CI: 0.45–0.63; Mother: OR = 0.71, 95% CI: 0.59–0.86), tooth-brushing frequency (OR = 0.35, 95% CI: 0.26–0.48), dental floss use (OR = 0.58, 95% CI: 0.41–0.83), sugar-containing drink consumption (OR = 2.11, 95% CI: 1.80–2.49), and dental visit (OR = 1.44, 95% CI: 1.19–1.74). Regarding the associated factors of calculus, the same model was used and significant variables including gender (OR = 1.32, 95% CI: 1.13–1.54), *hukou* (OR = 0.69, 95% CI: 0.59–0.82), family size (OR = 1.34, 95% CI: 1.12–1.59), parental educational level (Father: OR = 0.46, 95% CI: 0.39–0.54; Mother: OR = 0.65, 95% CI: 0.59–0.82), tooth-brushing frequency (OR = 0.57, 95% CI: 0.42–0.78), dental floss use (OR = 0.66, 95% CI: 0.48–0.90) and sugar-containing drink consumption (OR = 1.30, 95% CI: 1.11–1.53) are presented in Table 5. Tables 3 and 4 also displayed the parameters of the fixed and random effects in the two-level logistic regression models and their average intercepts. Finally, the percentage variance explained by the contextual level was 3.13% for gingival bleeding model and 2.27% for calculus model.

**Table 4:** Selected variables using training data shown to be associated with gingival bleeding by multi-level logistic regression analyses (*n* = 3394)

Variables	Categories	Null model	Model with individual and contextual variables	
			OR (95% CI)	***p***
**Fixed effect**				
**Individual level**				
***hukou***			1	
	Rural		**0.61 (0.52, 0.72)**	**< 0.01**
**Family size**	Urban		1	
	One child		**1.41 (1.19, 1.68)**	**< 0.01**
**Father’s educational level**	More than one child		1	
	Middle school or lower ^a^		**0.53 (0.45, 0.63)**	**< 0.01**
**Mother’s educational level**	More than middle school^b^		1	
			**0.71 (0.59, 0.86)**	**< 0.01**
**Brushing frequency (times/day)**			1	
			**2.03 (1.46, 2.82)**	**< 0.01**
			0.93 (0.70, 1.23)	0. 60
			**0.35 (0.26, 0.48)**	**< 0.01**
**Dental floss use**			1	
			**0.58 (0.41, 0.83)**	**< 0.01**
**Sugar-containing soft drink/Pop/ milk/yogurt/tea/coffee/water**			1	
			**2.11 (1.80, 2.49)**	**< 0.01**
**Dental visit (previous year)**			1	
			**1.44 (1.19, 1.74)**	**< 0.01**
**Random effect**				
**Contextual level variance (SE**^**c**^**)**		0.20 (0.12)	0.13 (0.08)	0.10
**AIC**^**d**^		4562.8	4051.4	
**Average intercept**		−0.173	−0.02246	
**VPC**^**e**^		0.0469	0.031252	

*Abbreviations*: ^a^Middle school or lower, ≤9 years; ^b^More than middle school, > 9 years; ^c^*SE* standard error, ^d^*AIC* Akaike Information Criteria, ^e^*VPC* variance partitioning coefficient attributable to the second level (regions)

**Table 5:** Selected variables using training data shown to be associated with dental calculus, evaluated by multi-level logistic regression analyses (n = 3394)

Variables	Categories	Null model	Model with individual and contextual variables	
			OR (95% CI)	***p***
**Fixed effect**				
**Individual level**				
**Gender**	Female		1	
	Male		**1.32 (1.13, 1.54)**	**< 0.01**
***hukou***	Rural		1	
	Urban		**0.69 (0.59 0.82)**	**< 0.01**
**Family size**	One child		1	
	More than one child		**1.34 (1.12, 1.59)**	**< 0.01**
**Father’s educational level**	Middle school or lower ^a^		1	
	More than middle school^b^		**0.46 (0.39 0.54)**	**< 0.01**
**Mother’s educational level**	Middle school or lower ^a^		1	
	More than middle school^b^		**0.65 (0.59 0.82)**	**< 0.01**
**Brushing frequency (times/day)**	Never		1	
	Sometimes		**1.76 (1.21, 2.54)**	**< 0.01**
	One		1.04 (0.771.42)	0.79
	≥Twice		**0.57 (0.42 0.78)**	**< 0.01**
**Dental floss use**	Never		1	
	Yes		**0.66 (0.48 0.90)**	**0.01**
**Sugar-containing soft drink/Pop/ milk/yogurt/tea/coffee/water**	≤1/week		1	
	> 1/week		**1.30 (1.11 1.53)**	**< 0.01**
**Dental visit (last year)**	No		1	
	Yes		1.15 (0.96 1.39)	0.14
**Frequency of dental curricula** **(last semester)**	0		1	
	≥1		0.85 (0.67, 1.07)	0.16
**Random effect**				
**Contextual level variance (SE**^**c**^**)**		0.065 (0.42)	0.12 (0.07)	0.11
**AIC**^**d**^		4277.8	3985.3	
**Average intercept**		0.715	1.06365	
**VPC**^**e**^		0.01438	0.022693	

*Abbreviations*: ^a^Middle school or lower: ≤9 years; ^b^More than middle school: >9 years; ^c^*SE* standard error, ^d^*AIC* Akaike Information Criteria, ^e^*VPC* variance partitioning coefficient

Based on these models with the average intercepts and coefficients of the variables before, area under the curve (AUC) values were calculated from the receiver operating characteristic (ROC) curves for gingival bleeding and calculus deposits (Table 6). As to the model of gingival bleeding, the estimated AUC values were 0.73 (95% CI: 0.72, 0.75) for the training data and 0.75 (95% CI: 0.73, 0.78) after applying the model by using the validation data. For the model of calculus deposits, the estimated AUC values were 0.68 (95% CI: 0.66, 0.70) for the training data and 0.67 (95% CI: 0.63, 0.69) for the validation data. Table 6 showed the performance values for all models, and the optimal cut-off values for the probabilities were calculated using maximized Youden indices. Based on these optimal cut-off values for all samples in the gingival bleeding models, the sensitivity, specificity, positive predictive value (PPV), negative predictive value (NPV), Youden index and predicted prevalence were determined.

**Table 6:** Summary findings of models in gingival bleeding and calculus

Performance Measures	Predictive model with total samples (%)	Predictive model with training data (%)	Predictive model with validation data (%)
**Gingival bleeding**			
Sensitivity	69.05 (67.08, 71.02)	68.77 (66.48, 71.07)	69.85 (66.00, 73.71)
Specificity	66.75 (64.87, 68.63)	66.19 (64.02, 68.36)	68.48 (64.73, 72.24)
PPV^a^	64.4 7 (62.50, 66.44)	63.54 (61.25, 65.83)	67.26 (63.39, 71.13)
NPV^b^	71.17 (69.30, 73.04)	71.22 (69.07, 73.37)	71.02 (67.29, 74.76)
Accuracy	67.82 (66.46, 69.18)	67.38 (65.81, 68.96)	69.14 (66.45, 71.83)
AUC^c^	73.81 (72.38,75.25)	73.29 (71.63,74.96)	75.34 (72.53, 78.15)
Predicted prevalence	49.94 (48.49, 51.40)	53.86 (52.18, 55.34)	49.96 (47.04, 52.87)
Youden index	35.93 (33.20, 38.65)	34.97 (31.81, 38.12)	38.34 (32.95, 43.72)
**Dental calculus**			
Sensitivity	67.12 (996, 68.79)	67.83 (65.91, 69.78)	65.02 (61.65, 68.39)
Specificity	59.43 (56.94, 61.91)	59.96 (57.11, 62.82)	57.73 (52.65, 62.82)
PPV^a^	77.01 (75.40, 78.61)	77.15 (75.31, 79.00)	76.57 (73.32, 79.82)
NPV^b^	47.16 (44.91,49.42)	48.3 3 (45.76, 50.90)	43.72 (39.28, 48.17)
Accuracy	64.57 (63.18, 65.97)	65.20 (63.60, 66.81)	62.69 (59.87, 65.51)
AUC^c^	67.83 (66.19, 69.46)	68.27 (66.39, 70.16)	66.55 (63.29, 69.28)
Predicted prevalence	58.34 (56.91 59.78)	58.54 (56.89, 60.20)	57.73 (54.86, 60.62)
Youden index	26.54 (23.54, 29.54)	27.80 (24.36, 31.24)	22.75 (16.65, 28.86)

*Abbreviations*: ^a^*PPV* positive predictive value, ^b^*NPV* negative predictive value, ^c^*AUC* area under the receiver operating characteristic (ROC) curve

## Discussion

This was a cross-sectional study included a relatively large sample size (*N* = 4525) to assess the prevalence of gingival bleeding and dental calculus in western Chinese adolescents. The study found high prevalence of both gingival bleeding and calculus, 46.63% of participants showed gingival bleeding, and 66.94% had calculus deposits. Here multistage sampling was used in this study, which was considered as a more accurate sampling [35, 36]. It is also a convenient and effective way for finding the survey sample and is particularly suitable for our study, which needed a large sample size [35, 36]. A higher prevalence of calculus was calculated than that of gingival bleeding. These results are contrast with the findings of previous studies [4, 37]. The reason may be the different qualities of water in western China, with high hardness and much salinity [38, 39]. Therefore, the effect of water supply on the oral health is needed to explore in the further study.

In the present study, multi-level models were used to correctly specify the data structure and to demonstrate the significant variables of gingival bleeding and calculus. As screening approach for assessing the associated factors of gingival bleeding and calculus, adolescents could be encouraged to modify bad behaviors to decrease the prevalence. For simple validation method, we randomly divided our study population into three-quarters and one-quarter samples used in the previous studies [20, 40]. Simple random sample was used to select the training data and validation data. Actually, the dataset selection should be considered its multilevel structure [41]. If this is not done, the analysis may give incorrect or potentially misleading results. Because the hierarchical datasets may be close to flat and candidate models can be swamped by random fluctuations [41]. Therefore, we confirmed the distribution of the samples in 6 regions were similar, and no significant bias existed. Table 6 showed the summary findings of our models [20]. Among, Youden’s index provides a measure of validity by considering both sensitivity and specificity [42]. Here the low values of Youden indices suggested the low predictive capacity of the models. The results also implied the models could only be used as a first-line screening tool. Regular dental visit is still needed. Previous study said ethnical and methodological differences would influence the predictive capacity [42]. In addition, this epidemiological survey included only 6 regions. A small region size cannot produce valid and accurate estimates for multi-level logistic regression models [43]. Further we will increase the regions and adolescents included. And factors in questionnaire, especially contextual variables, should be added in the survey as well. Then the prediction models would obtain more accuracy.

From a conceptual perspective, the results contribute to our understanding of the determinants of gingival bleeding and calculus. The popularity of calculus was higher in male gender, however, there was little sex inequalities presented in the outcome of gingival bleeding. Previous studies confirmed that prevalence of gingivitis in puberty was correlated with elevation in systemic levels of the sex hormones (testosterone in boys and estradiol and progesterone in girls) [4, 6, 7]. Increased serum levels (testosterone in boys, and estradiol, progesterone in girls) are positively associated with high levels of *Prevotella (P.) intermedia* and *P. nigrescens* [6]. The age peak of gingivitis in girls (11–13 years) was earlier than that in boys (13–14 years) [4, 6, 7]. Therefore, the increased sex hormones in girls may interfere the results. Along with gender, age and income, *hukou* is quite a common variable on individual behavior in social and economic studies in China [44, 45]. It was proved to be associated with dental health. But previous studies were focused on its impact on dental caries [46, 47]. In this study, the periodontal status was better in urban *hukou* than that in rural *hukou.* The reason might be people with rural *hukou* didn’t have full access to oral-related public health services funded by the tax revenue [46, 47].

Several characteristics, broadly categorized as child-level, family-level and community-level, could influence the periodontal diseases in adolescents [48, 49]. Family-level characteristics affect both the child-level factors, such as self-care practices and service utilization, and periodontal disease status [50, 51]. This is because parents and adolescents share similar cariogenic and periodontal pathogenic microbiota [52, 53]. The results of our analyses suggested that sociodemographic characteristics of adolescents’ family at 12 years of age, as well as family environment, were important determinants of the occurrence of gingival bleeding and calculus in adolescents. In this study, the only child had lower prevalence of gingival bleeding and calculus than child with siblings. The results were similar with many previous studies that proved less adolescent number in one family was easier to have better periodontal condition [54, 55]. In addition, our study observed a positive impact of higher level of parental education on the gingival status of adolescents, and mothers’ and fathers’ education levels, respectively, could be used as a predictor of gingival bleeding and calculus.

As to the self-care practices and habits, unhealthy diet behaviors (high carbohydrate diet) and bad hygiene care (less brushing frequency per day) increased the prevalence of gingival bleeding and calculus. However, no significant difference was found between adolescents brushing once/day and those did not brush at all. This thought-provoking point surprised us. The potential reasons to interpret this phenomenon are as following: first, gingivitis is caused by multiple factors [54], including not only brushing frequency, but also duration of brushing, brushing method, and the type of dentifrice [55]. Second, people with irregular health habits, such as meals, tooth-brushing and bedtime, are more prone to disease [56, 57]. Third, reporting bias and undercounting of errors were unavoidable in this survey.

The periodontal status of the included adolescents in this survey was unsatisfactory, and most of them had calculus. However, a somewhat high number of adolescents claimed to brush their teeth at least once a day. This inconsistency between periodontal health and reported dental hygiene practice may be a result of the adolescents having not acquired the proper brushing techniques. Motivation to implement the instructions given for oral health care and regular enforcement are essential in promoting adolescents’ oral health [58]. In China, dental curricula are not enforced and only provided in certain schools in the health education textbook [59]. Here, the popularity of dental curricula and the relationship between this intervention and oral health were explored. Dental curricula showed limited impact on the oral health improvement, which suggested that better and more effective oral health education was essential.

Only 20% of the students had visited a dentist during the last 12 months, which was a lower proportion than that found in the other surveys [60]. However, a higher gingivitis rate was found in the adolescents who had visited dentists. The trend was consisted with previous studies [35, 36]. This might be due to the common problem-oriented dental-care-seeking behavior in China. It was plausible that adolescents were brought to visit dentists for pain and infection [35, 36]. The limited availability of dental-care resources might be another reason. The dentist to population ratio is around 1:30,000 in China and the number of dentists is even fewer in rural regions [61].

Some limitations need to be analyzed in this investigation. This was a cross-validation analysis, an external validation was not possible because of the limited numbers of variables obtained from other screening database. In addition, the questionnaires were completed by the participants; thus, certain limitations were unavoidable, such as response bias, reporting bias and undercounting of errors. Moreover, more contextual variables that reflect the inequitable distribution of dental-care resources and oral health coverage, water supply and utilization of dental services need to be assessed. At last, this epidemiological survey included only 6 regions in Sichuan Province based on a complex sample design. A small region size may hardly represent the whole province’s population, and generate biased estimates. Because of these limitations, our results should be interpreted with caution.

The findings from this study can serve as a warning sign for adolescents and policy makers in western China. Oral health education and practice should be strengthened, especially for adolescents with high risk. Individual, parents and educators should be instructed about the prevention and management of gingival disease. And oral health educational programs for parents are also imperative. Tackling the inequitable distribution of dental care resources is very important. Due to lack of dentists in some regions, the cultivation of a community-based dentist may be a priority, which can improve the adolescents’ access to dental care.

## Conclusion

Gingival bleeding and calculus were prevalent among 12-year-old western Chinese adolescents. Unhealthy diet behaviors and insufficient hygiene care were related to the high prevalence. Individual, family, and school should be instructed about the prevention and management for improving the gingival status of adolescents. Moreover, regular dental visit is also recommended.

## Data Availability

The datasets used and analyzed during the current study are available from the corresponding author on reasonable request.

## Abbreviations

SDH: Social determinants of health
CSDH: Commission on social determinants of health
WHO: World Health Organization
PPS: Probability-proportional-to-size
BOP: Bleeding on probing
VPC: Variance partition coefficient
ROC: Receiver operating characteristic
AUC: Area under the ROC curve
AIC: Akaike information criterion
CPI probe: Community periodontal index probe
SE: Standard error
PPV: Positive predictive value
NPV: Negative predictive value
